# CAR-T Cells Therapy in Glioblastoma: A Systematic Review on Molecular Targets and Treatment Strategies

**DOI:** 10.3390/ijms25137174

**Published:** 2024-06-29

**Authors:** Edoardo Agosti, Alexandru Garaba, Sara Antonietti, Tamara Ius, Marco Maria Fontanella, Marco Zeppieri, Pier Paolo Panciani

**Affiliations:** 1 Division of Neurosurgery, Department of Medical and Surgical Specialties, Radiological Sciences and Public Health, University of Brescia, Piazza Spedali Civili 1, 25123 Brescia, Italy; edoardo_agosti@libero.it (E.A.);; 2 Neurosurgery Unit, Head-Neck and NeuroScience Department, University Hospital of Udine, p.le S. Maria della Misericordia 15, 33100 Udine, Italy; 3 Department of Ophthalmology, University Hospital of Udine, p.le S. Maria della Misericordia 15, 33100 Udine, Italy

**Keywords:** glioblastoma, immunotherapy, molecular targets, CAR T cell, systematic reviews

## Abstract

The most common primary brain tumor is glioblastoma (GBM), yet the current therapeutic options for this disease are not promising. Although immunotherapeutic techniques have shown poor success in GBM thus far despite efforts, new developments provide optimism. One of these developments is chimeric antigen receptor (CAR)-T cell treatment, which includes removing and genetically modifying autologous T cells to produce a receptor that targets a GBM antigen before reintroducing the cells into the patient’s body. A number of preclinical studies have produced encouraging results, which have led to the start of clinical trials assessing these CAR-T cell treatments for GBM and other brain tumors. Although results in tumors such as diffuse intrinsic pontine gliomas and lymphomas have been promising, preliminary findings in GBM have not produced any clinical benefits. The paucity of particular antigens in GBM, their inconsistent expression patterns, and the possible immunoediting-induced loss of these antigens after antigen-targeted therapy are some possible causes for this discrepancy. The goal of this systematic literature review is to assess potential approaches for creating CAR-T cells that are more effective for this indication, as well as the clinical experiences that are already being had with CAR-T cell therapy in GBM. Up until 9 May 2024, a thorough search was carried out across the three main medical databases: PubMed, Web of Science, and Scopus. Relevant Medical Subject Heading (MeSH) terms and keywords associated with “glioblastoma”, “CAR-T”, “T cell therapy”, “overall survival”, and “progression free survival” were employed in the search approach. Preclinical and clinical research on the application of CAR-T cells as a therapeutic approach for GBM are included in the review. A total of 838 papers were identified. Of these, 379 articles were assessed for eligibility, resulting in 8 articles meeting the inclusion criteria. The included studies were conducted between 2015 and 2023, with a total of 151 patients enrolled. The studies varied in CAR-T cell types. EGFRvIII CAR-T cells were the most frequently investigated, used in three studies (37.5%). Intravenous delivery was the most common method of delivery (62.5%). Median OS ranged from 5.5 to 11.1 months across the studies. PFS was reported in only two studies, with values of 7.5 months and 1.3 months. This systematic review highlights the evolving research on CAR-T cell therapy for GBM, emphasizing its potential despite challenges. Targeting antigens like EGFRvIII and IL13Rα2 shows promise in treating recurrent GBM. However, issues such as antigen escape, tumor heterogeneity, and immunosuppression require further optimization. Innovative delivery methods, combination therapies, and personalized approaches are crucial for enhancing CAR-T cell efficacy. Ongoing research is essential to refine these therapies and improve outcomes for GBM patients.

## 1. Introduction

Glioblastoma (GBM) is the most common and aggressive type of glial tumor, representing over 50% of all primary brain tumors in the United States, with an annual incidence rate of about 3 per 100,000 people [1]. Despite rigorous treatment plans, which include surgery, chemotherapy, and radiation, the median overall survival (OS) for GBM patients is only around 15 months, and the 5-year OS rate is less than 10%. The difficulty in treating GBM arises from a small population of glioblastoma stem cells (GSCs) that resist therapy, and the intricate inter- and intra-tumor heterogeneity within the tumor microenvironment (TME) [2]. These GSCs play a crucial role in tumor recurrence and resistance due to their strong DNA repair capabilities, multi-drug resistance properties [3], and ability to evade the immune system. Additionally, the GBM TME promotes tumor growth and creates an immunosuppressive environment, making treatment even more challenging.

Chimeric antigen receptor (CAR)-T cell therapy, an innovative immunotherapeutic method, has shown potential in overcoming these obstacles. CAR-T cells are designed to recognize and attack specific antigens on GBM cells, providing a highly tailored and potent treatment option. This therapy involves modifying a patient’s T cells to express CARs that specifically target antigens associated with GBM, thereby boosting the immune system’s capacity to identify and eliminate tumor cells [4]. While CAR-T cell therapy has been highly successful in treating hematologic cancers, its use in GBM is still in the preliminary stages, with ongoing clinical trials investigating its effectiveness and safety. This systematic literature review focuses exclusively on the role of CAR-T cells in GBM treatment. We will delve into the molecular mechanisms underlying CAR-T cell therapy, evaluate current clinical trial outcomes, and discuss the potential advantages and challenges associated with this approach. By synthesizing the latest research, this review aims to provide a comprehensive overview of CAR-T cell therapy in GBM, highlighting its potential to revolutionize the treatment landscape for this cancer.

## 2. Materials and Methods

### 2.1. Literature Review

The systematic review adhered to the guidelines outlined in the Preferred Reporting Items for Systematic Reviews and Meta-Analysis (PRISMA) [5]. Two researchers (E.A. and A.G.) conducted a thorough and methodical literature exploration across PubMed, Web of Science, and Scopus databases. The initial search was executed on 10 April 2024, with subsequent updates made on 9 May 2024. A comprehensive search strategy was formulated using a combination of relevant keywords such as “glioblastoma”, “CAR-T”, “T cell therapy”, “overall survival”, and “progression free survival”, employing both AND and OR operators. Retrieval of studies utilized Medical Subject Heading (MeSH) terms and Boolean logic: (“glioma” OR “glioblastoma” OR “GBM”) AND (“CAR-T” OR “CAR-T therapy” OR “chimeric antigen receptor” OR “genetically modified T”) AND (“outcomes” OR “prognosis” OR “progression free survival” OR “overall survival”).

Additional relevant articles were identified through a thorough examination of references cited in selected papers. All studies were included based on specific criteria: (1) written in English; (2) clinical trials, encompassing single-arm or double-arm studies, including randomized controlled trials or non-randomized controlled trials; (3) focusing on immunotherapy strategies for glioblastoma (GBM) using CAR-T cells, either as monotherapy or in combination with chemotherapy (CT) and/or radiotherapy (RT); (4) studies that analyzed at least overall survival (OS) and progression-free survival (PFS) as outcomes. Exclusion criteria consisted of (1) editorials, case reports, case series, cohort studies, literature reviews, and meta-analyses; (2) studies lacking clear methodological descriptions or results; (3) studies without reported data on PFS or OS.

The identified studies were imported into Endnote X9, and duplicate entries were eliminated. Subsequently, two independent researchers (E.A. and A.G.) reviewed the results against the inclusion and exclusion criteria. Any discrepancies were resolved by a third reviewer (P.P.P.). Eligible articles then underwent full-text screening.

### 2.2. Data Extraction

Each study was analyzed to extract the following details: authorship, publication year and journal, title, clinical trial phase and name, patient count, diagnosis, duration of follow-up, CAR-T therapy specifics, method of drug administration, and study outcomes.

### 2.3. Outcomes

Our primary outcomes were OS and PFS related to CAR-T cell therapy.

### 2.4. Risk of Bias Assessment

**Figure 1:**
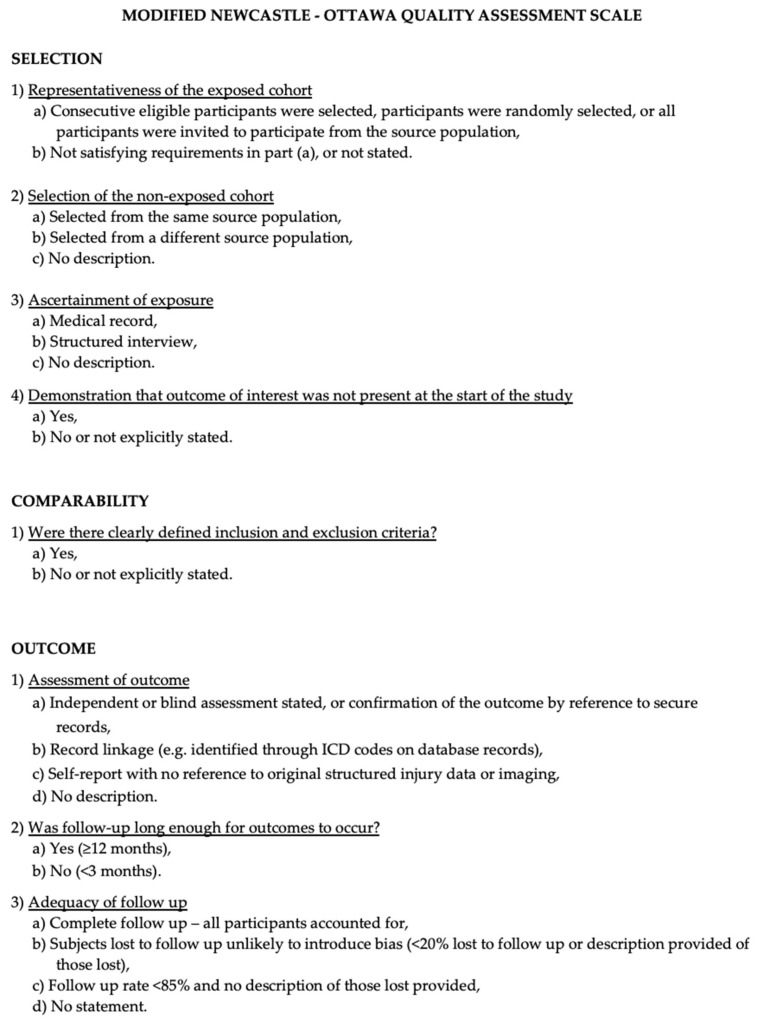
The Modified NOS.

The Newcastle-Ottawa Scale (NOS) [6] was used to assess the quality of the included studies. Quality assessment was performed by assessing the selection criteria, comparability of the study, and outcome assessment. The ideal score was 9. Higher scores indicated better quality of studies. Studies receiving 7 or more points were considered high-quality studies. Two authors (E.A. and P.P.P.) performed the quality assessment independently. When discrepancies arose, papers were re-examined by the third author (Figure 1).

### 2.5. Statistical Analysis

Descriptive statistics were reported, including ranges and percentages. All statistical analyses were performed using the R statistical package v3.4.1 (http://www.r-project.org).

## 3. Results

### 3.1. Literature Review

**Figure 2:**
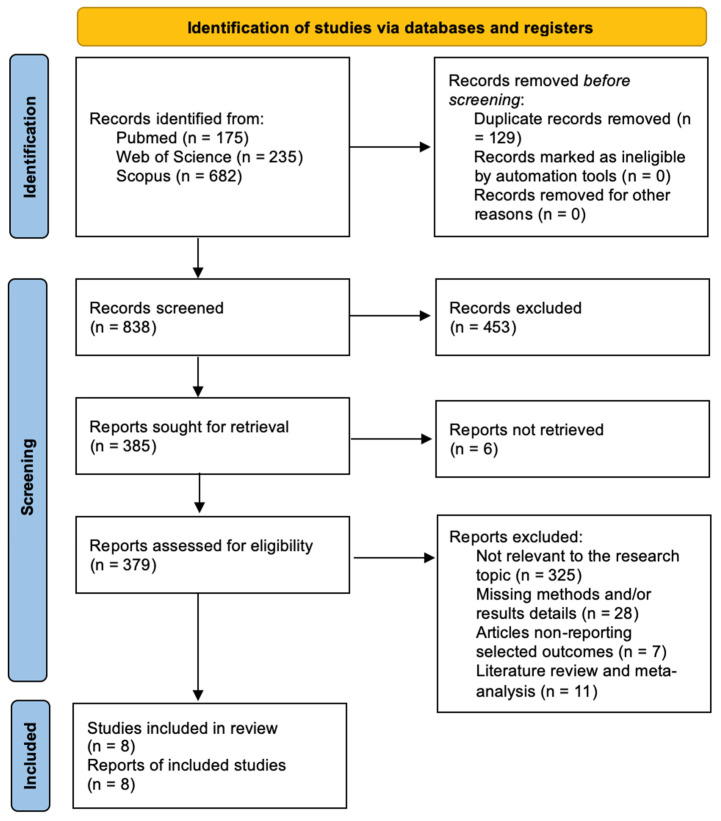
PRISMA flow chart.

After eliminating duplicates, a total of 838 papers were initially identified. Following review of titles and abstracts, 385 articles were selected for full-text assessment. Among these, 379 articles met the eligibility criteria, resulting in 8 included studies. The remaining 371 articles were excluded for various reasons: (1) lack of relevance to the research topic (325 articles), (2) absence of reported outcomes of interest (7 articles), (3) being systematic reviews or meta-analyses (11 articles), and (4) insufficient methodological or results detail (28 articles). All studies included in the analysis reported at least one outcome measure for one or more patient groups studied. Figure 2 shows the flow chart according to the PRISMA statement.

**Figure A1:**
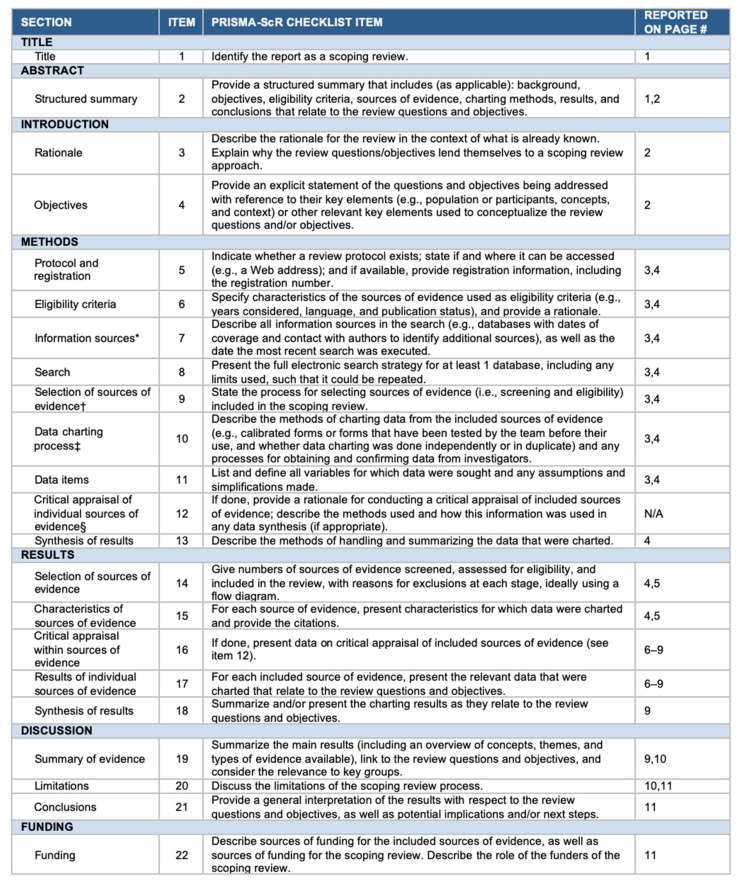
The PRISMA-ScR checklist. Abbreviations: JBI = Joanna Briggs Institute; PRISMA-ScR = Preferred Reporting Items for Systematic reviews and Meta-Analyses extension for Scoping Reviews. * Where sources of evidence (see second footnote) are compiled from, such as bibliographic databases, social media platforms, and Web sites. † A more inclusive/heterogeneous term used to account for the different types of evidence or data sources (e.g., quantitative and/or qualitative research, expert opinion, and policy documents) that may be eligible in a scoping review as opposed to only studies. This is not to be confused with information sources (see first footnote). ‡ The frameworks by Arksey and O’Malley (6) and Levac and colleagues (7) and the JBI guidance (4, 5) refer to the process of data extraction in a scoping review as data charting. § The process of systematically examining research evidence to assess its validity, results, and relevance before using it to inform a decision. This term is used for items 12 and 19 instead of “risk of bias” (which is more applicable to systematic reviews of interventions) to include and acknowledge the various sources of evidence that may be used in a scoping review (e.g., quantitative and/or qualitative research, expert opinion, and policy document).

Appendix A containing the PRISMA-ScR checklist is accessible for reference (Figure A1).

### 3.2. Data Analysis

A summary of the included studies is presented in Table 1.

All the studies included in this systematic review are clinical trials focusing on the application of CAR-T cell immunotherapy for GBM [7,8,9,10,11,12,13,14]. The publication years range from 2015 to 2023, with a total of 151 patients enrolled across these trials. One study was published in 2015, two in 2016, one in 2017, one in 2018, two in 2019, and one in 2023. All studies were Phase I trials, indicating the early stage of clinical research in this area.

The total number of patients enrolled in these trials was 151, with the majority of studies (62.5%) enrolling 10 or fewer patients each, and the remaining 37.5% enrolling more than 10 patients. All studies targeted recurrent GBM, reflecting the focus on treating this aggressive form of brain cancer.

Follow-up data were inconsistently reported across the studies, with only one study providing a median follow-up period of 24 months. The treatments investigated included various types of CAR-T cells: HER2 CMV-specific CAR-T cells, IL13(E13Y)-zetakine+ CD8+ CTL clones, IL13 Rα2-specific CAR-T cells, CAR-T-EGFRvIII+, EphA2-41BBζ T cells, and autologous GD2-specific 4SCAR-T cells. The most frequent treatment was EGFRvIII CAR-T cells, which were used in three studies (37.5%).

Regarding drug delivery methods, intravenous delivery was the most common, used in five studies (62.5%), while intracranial delivery was used in two studies (25%), and one study did not provide this information (12.5%).

The primary outcomes of interest were OS and PFS. Median OS (mOS) varied across the studies, with reported values of 11.1 months, 10.3 months, 7.5 months, 251 days (approximately 8.3 months), 5.5 months, 247 days (approximately 8.2 months), 6.9 months, and 10 months. Only two studies reported PFS, with one study showing a PFS of 7.5 months and another reporting 1.3 months [9,13].

In summary, all studies reviewed were Phase I clinical trials, primarily enrolling fewer than 10 patients each and targeting recurrent GBM. Intravenous delivery was the most common method, and mOS ranged from 5.5 months to 11.1 months. PFS was reported in only two studies. This comprehensive analysis underscores the early-phase nature of these trials, the small patient cohorts, and the varied approaches to treatment and delivery. Despite promising outcomes in some studies, further research with larger patient cohorts and longer follow-up periods is needed to establish the efficacy and safety of CAR-T cell therapies in GBM.

In Table 2 are listed the ongoing clinical trials. The ongoing clinical trials investigating CAR-T cell immunotherapy for GBM provide valuable insights into the evolving landscape of research in this area. These trials span from 2010 to 2023. The distribution of studies by publication year is as follows: 2023 (6 studies, 28.57%), 2022 (4 studies, 19.04%), 2021 (2 studies, 9.52%), 2020 (5 studies, 23.80%), 2019 (3 studies, 14.28%), and 2010 (1 study, 4.76%). They predominantly consist of Phase I trials, except for one study with an unspecified phase. In total, these trials encompass 564 patients, with the majority enrolling 31–40 patients (7 studies, 33.33%), followed by fewer than 11 patients (6 studies, 28.57%), 21–30 patients (3 studies, 14.28%), more than 40 patients (3 studies, 14.28%), and 11–20 patients (2 studies, 9.52%). All studies target glioma, with the majority focusing on recurrent GBM (76.19%). Follow-up durations vary across trials, with one study reporting a median follow-up period of 7.8 months. Various treatments are under investigation, with notable focus on B7-H3-targeted CAR-T cells (6 studies), *EGFRvIII* CAR-T cells (4 studies), and IL13 Rα2-targeted CAR-T cells (3 studies). Intracranial delivery methods, including intratumoral, intracerebroventricular, or intracavitary administration, are predominantly utilized, alongside intravenous delivery in some studies. The primary objectives across trials include safety, efficacy, tolerability, feasibility, dose-limiting toxicity (DLT), maximum tolerated dose (MTD), and OS. Safety assessment emerges as a common primary objective, followed by efficacy and feasibility evaluations. This comprehensive analysis highlights the diverse approaches and ongoing research efforts in Phase I trials of CAR-T cell therapies for GBM, with recurrent GBM being the primary focus of investigation.

## 4. Discussion

### 4.1. Molecular Mechanisms of CAR-T Cell Immunotherapy

CAR-T cell therapy represents a sophisticated immunotherapeutic approach aimed at harnessing the cytotoxic potential of T cells to target and eliminate tumor cells. The fundamental molecular mechanisms underlying CAR-T cell therapy involve the genetic modification of patient-derived T cells to express CARs specifically engineered to recognize tumor-associated antigens (TAAs) [15]. The CAR construct typically comprises three main domains: an extracellular antigen recognition domain, a transmembrane domain, and intracellular signaling domains. The extracellular domain is responsible for antigen recognition and typically consists of a single-chain variable fragment (scFv) derived from an antibody specific to the chosen TAA. This scFv enables CAR-T cells to bind specifically to surface antigens expressed on tumor cells. Following antigen recognition, the intracellular signaling domains initiate downstream signaling cascades that trigger T cell activation and effector functions. The primary signaling domain is derived from the CD3ζ chain of the T cell receptor (TCR) complex, which is essential for T cell activation upon antigen recognition. Additionally, co-stimulatory signaling domains, such as CD28 or 4-1BB (CD137), are often incorporated into the CAR construct to provide secondary signaling cues that enhance T cell proliferation, survival, and cytotoxic activity.

Upon binding to the target antigen expressed on tumor cells, CAR-T cells undergo activation, leading to the secretion of cytotoxic molecules such as perforin and granzyme, as well as the release of pro-inflammatory cytokines such as interferon-gamma (IFN-γ) and tumor necrosis factor-alpha (TNF-α). These effector mechanisms collectively result in the recognition and destruction of tumor cells by CAR-T cells [16].

Studies by June et al. (2018) [17] and Maude et al. (2014) [18] provide detailed insights into the design and engineering of CAR constructs, emphasizing the importance of optimizing antigen recognition and signaling domains to enhance CAR-T cell efficacy and specificity. Additionally, research by Guedan et al. (2018) [19] and Roybal et al. (2016) [20] explores innovative strategies for enhancing CAR-T cell functionality through the incorporation of novel co-stimulatory domains and genetic modifications. This aligns with the broader literature, which highlights the growing interest and investment in CAR-T cell therapy as a promising treatment modality for various malignancies, including hematological malignancies and solid tumors. Studies by June et al. (2018) [17] and Neelapu et al. (2018) [21] discuss the remarkable clinical efficacy of CAR-T cell therapy in patients with refractory B-cell malignancies, paving the way for further exploration of this approach in solid tumors such as GBM.

### 4.2. Targeted Therapies Proposed Using CAR-T Cells

In addition to understanding the molecular mechanisms underlying CAR-T cell therapy, it is crucial to evaluate the efficacy of targeted therapies proposed for GBM using CAR-T cells. Our study sheds light on ongoing clinical trials investigating CAR-T cell immunotherapy, particularly focusing on recurrent GBM, a highly aggressive form of brain cancer known for its resistance to conventional treatments. Various targeted antigens have been identified for CAR-T cell therapy in GBM, each with its unique molecular profile and therapeutic implications. Notable targets include B7-H3, EGFRvIII, and IL13Rα2, all of which are overexpressed on the surface of GBM cells, making them attractive candidates for CAR-T cell targeting.

B7-H3, also known as CD276, is a member of the B7 family of immune checkpoint molecules that regulate T cell responses. The overexpression of B7-H3 has been observed in various cancers, including GBM, where it contributes to tumor progression and immune evasion [22]. Preclinical studies have demonstrated the efficacy of B7-H3-targeted CAR-T cells in suppressing tumor growth and improving survival outcomes in vitro and in orthotopic and metastatic xenograft mouse models, which included patient-derived xenograft [23,24]. Clinical trials investigating the safety and efficacy of B7-H3-targeted CAR-T cell therapy in patients with recurrent GBM are currently underway, with promising early results reported.

EGFRvIII is a constitutively active mutant form of the EGFR protein that is frequently expressed in GBM but absent in normal tissues, making it an attractive target for CAR-T cell therapy [25]. Preclinical studies have shown that EGFRvIII-targeted CAR-T cells can effectively recognize and eliminate EGFRvIII-positive GBM cells in vitro [26]. Clinical trials evaluating the safety and efficacy of EGFRvIII-targeted CAR-T cell therapy in patients with GBM have demonstrated encouraging results, with some patients achieving durable responses and prolonged survival [10,12,13].

IL13Rα2 is a cell surface receptor overexpressed in a subset of GBM tumors, particularly in the mesenchymal subtype associated with poor prognosis [27]. IL13Rα2-targeted CAR-T cells have shown promising antitumor activity in preclinical models of GBM, leading to the initiation of clinical trials to evaluate their safety and efficacy in patients with IL13Rα2-positive GBM [28]. Early-phase clinical data suggest that IL13Rα2-targeted CAR-T cell therapy may induce tumor regression and improve survival outcomes in a subset of patients with recurrent GBM [8,9]. These studies highlight the potential of CAR-T cell therapy as a promising treatment modality for recurrent GBM, offering new hope for patients with this devastating disease.

The identification of predictive biomarkers for CAR-T cell therapy in GBM is another area of active research, aiming to improve patient selection and treatment outcomes. Biomarkers such as specific antigen expression levels on tumor cells, genetic and molecular profiles of both tumors and patients, and immune microenvironment characteristics are being explored. For instance, the presence of target antigens like EGFRvIII or IL13Rα2 on glioblastoma cells has been correlated with better responses to corresponding CAR-T cell therapies. Additionally, the genetic profiling of tumors to identify mutations or alterations that might influence CAR-T cell efficacy is under investigation. Immune profiling, including the assessment of immune cell infiltration and cytokine profiles within the tumor microenvironment, also offers potential as predictive biomarkers. These efforts aim to tailor CAR-T cell therapies to individual patients, maximizing therapeutic benefits while minimizing adverse effects. Further research and clinical validation are essential to establish reliable biomarkers that can guide clinical decision-making and optimize CAR-T cell therapy outcomes in GBM patients [12,13,25].

It must be remembered, however, that the development and clinical application of CAR-T cell therapy for GBM have not been without challenges, particularly concerning adverse effects that have led to the halting or failure of certain trials [3,7]. Notably, several Phase I trials have encountered severe adverse effects such as cytokine release syndrome (CRS), neurotoxicity, and on-target, off-tumor toxicity, which have raised significant safety concerns. For example, a trial targeting IL13Rα2 experienced severe neurotoxicity in a subset of patients, leading to its early termination. Another trial focusing on EGFRvIII-targeted CAR-T cells was halted due to instances of severe CRS, highlighting the need for more refined safety mechanisms [12,25]. These adverse events underscore the importance of developing robust strategies to mitigate toxicity, such as the incorporation of safety switches in CAR-T cell designs and improved patient monitoring protocols. By understanding the reasons behind these trial failures, future research can better navigate the complex safety landscape of CAR-T cell therapy, ultimately enhancing its therapeutic potential while minimizing risks [16].

Ongoing clinical trials investigating CAR-T cell therapy for GBM, underscore the importance of comprehensive safety and efficacy assessments in optimizing treatment outcomes. These trials build upon the foundational knowledge generated from preclinical studies and early-phase clinical trials, providing valuable insights into the feasibility and effectiveness of CAR-T cell therapy for GBM in real-world clinical settings.

### 4.3. Limitations and Future Perspectives

While CAR-T cell immunotherapy holds great promise for the treatment of GBM, several limitations and challenges remain to be addressed. One major limitation is the heterogeneity of GBM tumors, which can lead to variable responses to CAR-T cell therapy among patients. This heterogeneity is reflected in the diversity of antigens expressed by tumor cells, the genetic and molecular variability within and between tumors, and the differences in tumor microenvironments. The reviewed studies often had small patient cohorts, which limits the generalizability of their findings. Additionally, inconsistent follow-up durations across trials make it difficult to draw definitive conclusions about the long-term efficacy and safety of CAR-T cell therapies.

Another critical limitation is the variability in the findings of the studies analyzed. Differences in CAR-T cell constructs, delivery methods, patient selection criteria, and clinical endpoints contribute to the diverse range of outcomes reported. This variability complicates the ability to draw definitive conclusions and underscores the need for standardized methodologies in future research. The inconsistency in study designs and endpoints highlights the necessity for larger, more uniform trials to provide clearer insights into the efficacy and safety of CAR-T cell therapy for GBM.

The hostile tumor microenvironment of GBM, characterized by immunosuppressive factors and physical barriers such as the blood-brain barrier, poses significant challenges to the effective trafficking and infiltration of CAR-T cells into the tumor site. Moreover, the potential for on-target, off-tumor toxicity remains a concern, particularly when targeting antigens expressed at low levels in normal tissues. The reviewed studies highlighted instances of neurotoxicity and cytokine release syndrome, which underscore the need for improved safety mechanisms.

To overcome these limitations, future research efforts should focus on several key areas: (1) enhancing target specificity: identifying novel CAR-T cell targets that are highly specific to GBM cells while minimizing off-target effects is crucial. This includes the development of dual-targeting CAR-T cells that require the presence of two antigens for activation, thereby reducing the risk of attacking healthy tissues; (2) improving tumor infiltration: strategies to enhance the trafficking and infiltration of CAR-T cells into the GBM tumor microenvironment are needed. This could involve the use of adjuvant therapies that modulate the tumor microenvironment to be more conducive to CAR-T cell activity, such as immune checkpoint inhibitors or agents that disrupt the physical barriers of the tumor; (3) combining therapies: the development of combinatorial treatment approaches incorporating CAR-T cell therapy with other modalities such as immune checkpoint inhibitors, targeted therapies, and radiation therapy holds promise for enhancing therapeutic efficacy and overcoming resistance mechanisms; (4) optimizing CAR-T design: advancements in CAR-T cell engineering, including the incorporation of safety switches, optimization of CAR design, and the use of synthetic biology to control CAR-T cell functions, may further improve the safety profile and clinical outcomes of CAR-T cell therapy for GBM; (5) conducting larger, multicenter trials: to validate the efficacy and safety of CAR-T cell therapies, larger, multicenter clinical trials with standardized protocols and longer follow-up periods are essential. These trials should aim to include diverse patient populations to ensure the findings are broadly applicable [29,30].

## 5. Conclusions

This systematic review of the literature on CAR-T cell immunotherapy for glioblastoma reveals a dynamic and evolving landscape of research aimed at addressing the formidable challenges posed by this aggressive brain tumor. Molecular mechanisms underlying CAR-T cell therapy underscore the intricate interplay between engineered T cells and tumor cells, with emphasis on antigen recognition, activation, and cytotoxicity. Targeted therapies employing CAR-T cells, particularly those targeting B7-H3, EGFRvIII, and IL13Rα2, demonstrate promising results in preclinical and clinical settings, highlighting their potential as novel treatment modalities for recurrent glioblastoma. However, challenges such as antigen escape, tumor heterogeneity, and immunosuppressive microenvironment necessitate further optimization and refinement of CAR-T cell therapies. Integration of innovative delivery methods, combination therapies, and personalized approaches holds immense promise in harnessing the full potential of CAR-T cell immunotherapy for improving outcomes in patients with glioblastoma. Continued research efforts aimed at elucidating molecular mechanisms, optimizing treatment protocols, and addressing therapeutic limitations are imperative for advancing the field toward achieving meaningful clinical outcomes and ultimately improving the prognosis for patients with this disease.

## Figures and Tables

**Table 1:** Summary of the studies included in the systematic literature review.

Author	Year	Trial Name	Phase	Patients (N)	Diagnosis (Target Glioma)	Follow-Up (Months, Median Value)	Treatment	Drug Delivery	Endpoints (OS, PFS)
Ahmed et al. [7]	2015	NCT01109095	I	17	Recurrent GBM	N/A	HER2 CMV-specific CAR-T cells	Intravenous	OS: 11.1 mo from the first T-cell infusion and 24.5 mo from diagnosis. Three patients had no progression between 24 to 29 mo
Brown et al. [8]	2016	NCT00730613	I	3	Recurrent GBM	N/A	IL13(E13Y)-zetakine+ CD8+ CTL clones	Intracranial	mOS: 10.3 mo
Brown et al. [9]	2016	NCT02208362	I	82	Recurrent GBM	N/A	IL13 Rα2-specific CAR-T cells	Intracranial	PFS: 7.5 mo
O’Rourke et al. [10]	2017	NCT02209376	I	10	Recurrent GBM	N/A	CAR T-EGFRvIII+	Intravenous	mOS: 251 d
Lin et al. [11]	2018	NCT03423992	I	3	Recurrent GBM	N/A	EphA2-41BBζ T cells	Intravenous	mOS: 5.5 mo
Wang et al. [12]	2019	N/A	N/A	10	Recurrent GBM	N/A	EGFRvIII CAR-T cells	N/A	mOS: 247 d
Goff et al. [13]	2019	NCT01454569	I	18	Recurrent GBM	N/A	Autologous EGFRvIII-specific CAR-T cells	Intravenous	mOS: 6.9 mo; mPFS: 1.3 mo
Liu et al. [14]	2023	NCT03170141	I	8	Recurrent GBM	24	Autologous GD2-specific 4SCAR-T cells	Intravenous or directly to the tumor location	mOS: 10 mo

Abbreviations: CAR-T = chimeric antigen receptor T cells; GBM = glioblastoma; N/A = not available; OS = overall survival; PFS = progression free survival.

**Table 2:** Ongoing clinical trials.

Principal Investigator	Year	Trial Name	Phase	Status	Patients (N)	Diagnosis (Target Glioma)	Treatment	Drug Delivery	Primary Objectives
Badie	2010	NCT01082926	I	Completed	6	Recurrent malignant glioma	GRm13Z40-2 (Allogeneic CD8+ Cytolitic T-Cell Line Genetically Modified to Express the IL 13-Zetakine and HyTK and to be Resistant to Glucocorticoids) in Combination With Interleukin-2	Intratumoral	Safety
Badie	2019	NCT04003649	I	Recruiting	60	Recurrent GBM	IL13 Rα2-targeted CAR-T cells with or without nivolumab and ipilimumab	Intravenous	AEs; DLT; feasibility; OS
Chen	2019	NCT04045847	I	Unknown	31	Recurrent GBM	CD147-CART	Intracavity	Safety; Tolerance; Efficacy
O’Rourke	2019	NCT03726515	I	Completed	7	GBM (unmethylated MGMT, EGFRvIII+)	CART-EGFRvIII + Pembrolizumab	NA	Safety; tolerability
Mackall	2020	NCT04196413	I	Recruiting	54	*H3K27M*-mutated DIPG of the brainstem, or *H3K27M*-mutated DMG of the spinal cord	*GD2* CAR-T cells; Fludarabine; Cyclophosphamide	Intravenous	mOS: 7.8 mo
Omer	2020	NCT04099797	I	Recruiting	34	GD2-expressing newly diagnosed DMG (DIPG or HGG)	*C7R-GD2*.CART cells	Intracerebroventricularly	Efficacy
Badie	2020	NCT04214392	I	Recruiting	36	Recurrent GBM	Chlorotoxin (EQ)-CD28-CD3zeta-CD19t-expressing CAR T-lymphocytes	Intracerebroventricularly	Feasibility; safety
Reinikainen	2020	NCT05063682	I	Unknown	10	Leptomeningeal disease from glioblastoma	EGFRvIII-CAR-T cells	Intracerebroventricular	Feasibility; safety
Kuo	2020	NCT04717999	NA	Unknown	20	Recurrent GBM	NKG2D CAR-T cell	Intracerebroventricular injection through an Ommaya catheter	DLT
Feldman	2021	NCT04661384	I	Recruiting	30	leptomeningeal disease from glioblastoma, ependymoma, or medulloblastoma	IL13 Rα2-targeted CAR-T cells	Intracerebroventricular	AEs; OS
Xu	2021	NCT05131763	I	Unknown	3	Hepatocellular carcinoma, GBM, medulloblastoma and colon cancer	NKG2D CAR-T cells	NA	AEs
Zhang	2022	NCT04385173	I	Recruiting	12	Recurrent GBM	B7-H3-targeted CAR-T cells with temozolomide	Intratumoral/intracerebroventricular	AEs; MTD; OS; PFS
Cheng	2022	NCT05366179	I	Recruiting	36	Relapsed/refractory GBM	B7-H3 CAR-T cells	Intraventricular infusion	AEs
Zhang	2022	NCT05241392	I	Recruiting	30	Recurrent GBM	B7-H3 CAR-T cells	Intracranial tumor resection cavity or ventricular system using an Ommaya device	Incidence of AEs; DLT
Mackall	2022	NCT05474378	I	Recruiting	39	Recurrent GBM	B7-H3 CAR-T cells	ICV or both ICV and intratumorally (IT)	Number of successful manufacturing product (B7-H3CART) that met minimum assigned dose level range; MTD or Recommended phase 2 dose
Zhang	2023	NCT04077866	I/II	Recruiting	40	Recurrent GBM	B7-H3-targeted CAR-T cells with or without temozolomide	Intratumoral/intracerebroventricular	OS; PFS
Xuejun	2023	NCT05802693	I	Not yet recruiting	22	Recurrent GBM	EGFRvIII CAR-T cells	Infusion with Omaya capsule?	Incidence of AEs; DLT
Bagley	2023	NCT05168423	I	Recruiting	6	Recurrent GBM	EGFR-IL13Rα2 CAR-T cells	Intrathecal	Incidence of AEs; DLT
Doyle	2023	NCT05835687	I	Recruiting	36	Relapsed/refractory non-brainstem primary CNS tumors and brainstem high-grade neoplasms	B7-H3 CAR-T cells	CNS reservoir catheter	MTD
Litten	2023	NCT05627323	I	Recruiting	42	Recurrent GBM	CHM-1101 CAR-T cells	Intracavitary/intratumoral and intraventricular	Incidence of AEs; DLT
Zhang	2023	NCT05577091	I	Recruiting	10	Recurrent GBM	IL7Rα modified CAR T lymphocytes	Intratumoral or intraventricular administration via Ommaya reservoir	N/A

Abbreviations: AE = adverse events; CAR-T = chimeric antigen receptor T cells; CNS = central nervous system; DIPG = diffuse intrinsic pontine glioma; DLT = dose limiting toxicity; DMG = diffuse midline glioma; GBM = glioblastoma; HGG = high grade glioma; MTD = maximum tolerated dose; N/A = not available; OS = overall survival; PFS = progression free survival.

## Data Availability

Data available in a publicly accessible repository.
